# Tuberculosis in hospitalized patients: clinical characteristics of patients receiving treatment within the first 24 h after admission*

**DOI:** 10.1590/S1806-37132014000300011

**Published:** 2014

**Authors:** Denise Rossato Silva, Larissa Pozzebon da Silva, Paulo de Tarso Roth Dalcin

**Affiliations:** Federal University of Rio Grande do Sul School of Medicine; and Pulmonologist. Department of Pulmonology, Porto Alegre Hospital de Clínicas, Porto Alegre, Brazil; Federal University of Rio Grande do Sul School of Nursing, Porto Alegre, Brazil; Federal University of Rio Grande do Sul School of Medicine; and Pulmonologist. Department of Pulmonology, Porto Alegre Hospital de Clínicas, Porto Alegre, Brazil

**Keywords:** Tuberculosis, Hospitalization, Time-to-treatment, Emergency medicine, Delayed diagnosis

## Abstract

**Objective:**

To evaluate clinical characteristics and outcomes in patients hospitalized for tuberculosis, comparing those in whom tuberculosis treatment was started within the first 24 h after admission with those who did not.

**Methods:**

This was a retrospective cohort study involving new tuberculosis cases in patients aged ≥ 18 years who were hospitalized after seeking treatment in the emergency room.

**Results:**

We included 305 hospitalized patients, of whom 67 (22.0%) received tuberculosis treatment within the first 24 h after admission ( ≤24h group) and 238 (88.0%) did not (>24h group). Initiation of tuberculosis treatment within the first 24 h after admission was associated with being female (OR = 1.99; 95% CI: 1.06-3.74; p = 0.032) and with an AFB-positive spontaneous sputum smear (OR = 4.19; 95% CI: 1.94-9.00; p < 0.001). In the ≤24h and >24h groups, respectively, the ICU admission rate was 22.4% and 15.5% (p = 0.258); mechanical ventilation was used in 22.4% and 13.9% (p = 0.133); in-hospital mortality was 22.4% and 14.7% (p = 0.189); and a cure was achieved in 44.8% and 52.5% (p = 0.326).

**Conclusions:**

Although tuberculosis treatment was initiated promptly in a considerable proportion of the inpatients evaluated, the rates of in-hospital mortality, ICU admission, and mechanical ventilation use remained high. Strategies for the control of tuberculosis in primary care should consider that patients who seek medical attention at hospitals arrive too late and with advanced disease. It is therefore necessary to implement active surveillance measures in the community for earlier diagnosis and treatment.

## Introduction

Tuberculosis remains a major public health problem worldwide. It is estimated that one third of the world population is infected with *Mycobacterium tuberculosis*. In 2011, 9 million new tuberculosis cases were estimated to have occurred worldwide, with 1.4 million deaths. Brazil ranks 22nd among the 22 countries with the highest reported incidence of tuberculosis, with 42 cases/100,000 population in 2011.^(^ ^1^ ^)^

Disease control in the community depends on early diagnosis and treatment. Although tuberculosis control programs recommend that the diagnosis of tuberculosis be made at primary health care clinics, most patients are diagnosed in hospitals.^(^ ^2^ ^,^ ^3^ ^)^ In Porto Alegre, Brazil, 39% of all tuberculosis patients have been diagnosed in hospitals.^(^ ^4^ ^)^

A previous study conducted in a university hospital in Porto Alegre^(^ ^5^ ^)^ showed that the median time elapsed between hospital admission and diagnosis of tuberculosis was 6 days, the major factors associated with delayed diagnosis being extrapulmonary disease and negative sputum smears. The emergency room has always been the gateway for such patients. 

In this context, it is important to analyze the characteristics of patients diagnosed with tuberculosis and receiving tuberculosis treatment within the first 24 h after hospital admission. We hypothesize that such patients do not need hospitalization, and that strategies for the screening and management of tuberculosis in the primary care setting can be developed on the basis of the present study, contributing to reducing the rates of emergency room treatment and the burden of hospitalization for tuberculosis. 

The objective of the present study was to analyze clinical characteristics and major outcomes in patients who were hospitalized for tuberculosis and who started tuberculosis treatment within the first 24 h after admission. 

## Methods

This was a retrospective cohort study of patients who were diagnosed with and hospitalized for tuberculosis after seeking treatment in the emergency room of the *Hospital de Clínicas de Porto Alegre* (HCPA, Porto Alegre *Hospital de Clínicas*), located in the city of Porto Alegre, Brazil. 

The study was approved by the local research ethics committee. The authors signed a data use agreement, protecting the confidentiality of patient information. 

The study population consisted of new tuberculosis patients who were diagnosed after seeking treatment in the HCPA emergency room. We included patients who were 18 years of age or older and who were identified as new cases of tuberculosis on the basis of consensus criteria.^(^ ^6^ ^)^ The diagnosis of pulmonary tuberculosis was based on the criteria established by the Third Brazilian Thoracic Association Guidelines on Tuberculosis^(^ ^6^ ^)^: a) positive Ziehl-Neelsen staining for AFB (two positive sputum smears); b) positive Ziehl-Neelsen staining for AFB (one positive sputum smear and one positive sputum culture for *M. tuberculosis*); c) positive Ziehl-Neelsen staining for AFB and radiological findings consistent with pulmonary tuberculosis; d) a single positive sputum culture for *M. tuberculosis*; or e) epidemiological, clinical, and radiological findings consistent with pulmonary tuberculosis, together with a favorable response to treatment with antituberculosis drugs. The diagnosis of extrapulmonary tuberculosis was based on clinical examination findings and ancillary test results (depending on the site of disease). The exclusion criteria were as follows: reported tuberculosis cases in which the diagnosis was subsequently changed; and cases of patients who had started treatment before hospitalization.

The patients were retrospectively identified on the basis of data obtained from individual tuberculosis report forms in the *Sistema de Informação de Agravos de Notificação* (SINAN, Brazilian Case Registry Database), and patient charts were reviewed. At our hospital, computerized physician order entry of antituberculosis drugs automatically generates the SINAN report form; therefore, we were able to identify all of the patients who started treatment. 

Patient charts were reviewed by the investigators, who completed a standardized questionnaire including the following items: demographic data (age, gender, race, and level of education); comorbidities; smoking status; alcohol use; injection drug use; use of immunosuppressive drugs; history of tuberculosis; clinical form of tuberculosis; symptoms at admission; diagnostic methods; treatment regimen used; HIV infection; time from admission to initiation of treatment; length of hospital stay; ICU admission; need for and duration of mechanical ventilation; outcome of hospitalization (discharge or death); and outcome after discharge (cure, treatment nonadherence, or death). Post-discharge data were obtained by reviewing patient charts, by searching the SINAN database, or by telephoning the outpatient clinics where patients were being followed. 

Data were entered into a Microsoft Excel^(r)^ 2010 spreadsheet, after which they were processed and analyzed with the Statistical Package for the Social Sciences, version 18.0 (SPSS Inc., Chicago, IL, USA). 

The study variables were analyzed descriptively. Quantitative data were presented as mean ± SD or as median (interquartile range). Qualitative data were expressed as number of cases and proportion. 

For statistical analysis, patients were divided into two groups: the ≤24h group, comprising those who received tuberculosis treatment within the first 24 h after hospital admission and the >24h group, comprising those who did not. 

For continuous variables, we used the independent sample t-test or the Mann-Whitney U test. For qualitative variables, we used the chi-square test (Yates' correction or Fisher's exact test being used when necessary). 

The non-collinear variables that reached significance (p < 0.01) in the univariate analysis were included in a stepwise forward conditional binary logistic regression model (adjusted for gender and age) for each outcome. 

All statistical tests were two-tailed, and the level of significance was set at 5%.

## Results

Between January of 2008 and January of 2011, 305 patients diagnosed with tuberculosis were included in the study. 

Table 1 shows the characteristics of the patients studied and a comparison between the ≤24h and >24h groups. The mean age was 42.0 ± 17.2 years, and most (64.6%) of the patients were male and White (75.4%). Of the 305 tuberculosis patients, 110 (36.1%) had pulmonary tuberculosis, 143 (46.9%) had extrapulmonary tuberculosis, and 52 (17.0%) had concomitant pulmonary and extrapulmonary disease. A total of 191 patients (62.6%) were HIV-positive. The mean length of hospital stay was 27.7 ± 21.8 days. None of the patients were discharged within the first 24 h after hospital admission, 6 (2%) were discharged 24-48 h after admission, and 9 (3%) were discharged 24-72 h after admission. The mean time elapsed between admission and initiation of treatment was 8.8 ± 10.8 days. Tuberculosis treatment was initiated on the same day as diagnosis. Therefore, the mean length of hospital stay after diagnosis and initiation of treatment was 18.9 ± 19.1 days. Although there was no difference between HIV-positive and HIV-negative patients regarding the length of hospital stay (p = 0.921), the hospital stay was longer in patients with one or more comorbidities than in those without comorbidities (29.3 ± 23.1 days vs. 23.2 ± 16.9 days; p = 0.030). 

**Table 1:** - Patient characteristics and comparison between the group of patients who received tuberculosis treatment within the first 24 h after hospital admission and that of those who did not.a

Characteristics	Total	Group ≤24h	Group >24h	p
	N = 305	n = 67	n = 238	
Age, years^b^	42.0 ± 17.2	38.5 ± 16.5	42.9 ± 17.5	0.067
Age > 60 years	42 (13.8)	7 (10.4)	35 (14.7)	0.428
Gender				
Male	197 (64.6)	34 (50.7)	163 (68.5)	0.011
Female	108 (35.4)	33 (49.3)	75 (31.5)	
Race				
White	230 (75.4)	48 (71.6)	182 (76.5)	0.516
Non-White	75 (24.6)	19 (28.4)	56 (23.5)	
Smoking status				
Nonsmoker	162 (53.1)	33 (49.3)	129 (54.2)	0.285
Former smoker	52 (17.0)	9 (13.4)	43 (18.1)	
Smoker	91 (29.8)	25 (37.3)	66 (27.7)	
Alcoholism	89 (29.2)	20 (29.9)	69 (29.0)	1.000
Illicit drug use	81 (26.6)	21 (31.3)	60 (25.2)	0.397
Form of tuberculosis				
Pulmonary tuberculosis	110 (36.1)	41 (61.2)	69 (29.0)	< 0.001
Extrapulmonary tuberculosis	143 (46.9)	14 (20.9)	129 (54.2)	< 0.001
Combined pulmonary and extrapulmonary tuberculosis	52 (17.0)	12 (17.9)	40 (16.8)	0.977
Symptoms				
Cough	132 (43.3)	43 (64.2)	89 (37.4)	< 0.001
Weight loss	151 (49.5)	40 (59.7)	111 (46.6)	0.080
Night sweats	74 (24.3)	24 (35.8)	50 (21.0)	0.019
Fever	190 (62.3)	39 (58.2)	151 (63.4)	0.523
Dyspnea	46 (15.1)	11 (16.4)	35 (14.7)	0.879
Chest pain	34 (11.1)	9 (13.4)	25 (10.5)	0.512
AFB-positive spontaneous sputum smear				
Yes	71 (23.3)	36 (53.7)	35 (14.7)	< 0.001
No	93 (30.5)	14 (20.9)	79 (33.2)	
No sputum	141 (46.2)	17 (25.4)	124 (52.1)	
Chest X-ray				
Cavitary disease	33 (10.8)	15 (22.4)	18 (7.6)	0.001
Consolidation	52 (17.0)	16 (23.9)	36 (15.1)	0.134
Pleural effusion	58 (19.0)	7 (10.4)	51 (21.4)	0.065
Normal	42 (13.8)	3 (4.5)	39 (16.4)	0.015
Miliary pattern	37 (12.1)	10 (14.9)	27 (11.3)	0.405
Comorbidities				
Diabetes mellitus	19 (6.2)	2 (3.0)	17 (7.1)	0.266
Chronic kidney disease	8 (2.6)	0 (0.0)	8 (3.4)	0.207
Transplantation	7 (2.3)	0 (0.0)	7 (2.9)	0.354
Chronic liver disease	6 (2.0)	1 (1.5)	5 (2.1)	0.744
Neoplasia	13 (4.3)	2 (3.0)	11 (4.6)	0.740
HIV	191 (62.6)	39 (58.2)	152 (63.9)	0.482
Any comorbidity	224 (73.4)	47 (70.1)	177 (74.4)	0.593
Length of hospital stay, days^b^	27.7 ± 21.8	19.6 ± 22.6	29.9 ± 21.1	0.001
ICU admission	52 (17.0)	15 (22.4)	37 (15.5)	0.258
Mechanical ventilation	48 (15.7)	15 (22.4)	33 (13.9)	0.133
Outcomes				
In-hospital mortality	50 (16.4)	15 (22.4)	35 (14.7)	0.189
One-year mortality	97 (31.8)	23 (34.3)	74 (31.1)	0.723
Cure	155 (50.8)	30 (44.8)	125 (52.5)	0.326

a Values expressed as n (%), except where otherwise indicated.

b Values expressed as mean ± SD

A total of 67 patients (22.0%) received tuberculosis treatment within the first 24 h after hospital admission. The proportion of males was higher in the >24h group than in the ≤24h group (68.5% vs. 50.7%; p = 0.011). The proportion of patients with pulmonary tuberculosis alone was higher in the ≤24h group than in the >24h group (61.2% vs. 29.0%; p < 0.001), whereas the proportion of patients with extrapulmonary tuberculosis alone was higher in the >24h group than in the ≤24h group (54.2% vs. 20.9%; p < 0.001). Cough was more common in the ≤24h group than in the >24h group (64.2% vs. 37.4%; p < 0.001), as were night sweats (35.8% vs. 21.0%; p = 0.019). The proportion of patients with AFB-positive spontaneous sputum smears was significantly higher in the ≤24h group than in the >24h group (53.7% vs. 14.7%; p < 0.001). The proportion of patients with routine chest X-rays showing cavitary disease was significantly higher in the ≤24h group than in the >24h group (22.4% vs. 7.6%; p = 0.001), whereas the proportion of patients with normal chest X-rays was significantly higher in the >24h group than in the ≤24h group (16.4% vs. 4.5%; p = 0.015). The total length of hospital stay was shorter in the ≤24h group than in the >24h group (19.6 ± 22.6 days vs. 29.9 ± 21.1; p = 0.001). There were no differences between the two groups of patients regarding the following outcomes: ICU admission; mechanical ventilation use; in-hospital mortality; one-year mortality; and cure rate (p > 0.05 for all).

Table 2 shows the binary logistic regression analysis of the characteristics associated with initiation of tuberculosis treatment within the first 24 h after hospital admission. Initiation of tuberculosis treatment within the first 24 h after admission was independently associated with being female (OR = 1.99; 95% CI: 1.06-3.74; p = 0.032) and with an AFB-positive spontaneous sputum smear (OR = 4.19; 95% CI: 1.94-9.00; p < 0.001). 

**Table 2:** - Binary logistic regression for characteristics associated with tuberculosis treatment initiation within the first 24 h after hospital admission.

Variable	b	Wald	Significance	OR	95% CI
Age	0.01	2.26	0.133	1.02	1.00-1.03
Female gender	0.69	4.60	0.032	1.99	1.06-3.74
Pulmonary tuberculosis	0.73	2.88	0.090	2.08	0.89-4.83
Extrapulmonary tuberculosis	0.04	0.005	0.942	1.04	0.36-3.00
Cough	0.34	0.94	0.333	1.40	0.71-2.76
Night sweats	0.41	1.30	0.254	1.50	0.75-3.00
AFB-positive spontaneous sputum smear	1.43	13.37	< 0.001	4.19	1.94-9.00
Cavitary disease	−0.10	0.05	0.828	0.90	0.36-2.26
Pleural effusion	−0.50	1.00	0.317	0.61	0.23-1.62
Normal chest X-ray	−0.57	0.63	0.428	0.57	0.14-2.31
Constant	−0.57	0.26	0.611	0.57	-

## Discussion

This retrospective cohort study evaluated new cases of tuberculosis treated in the emergency room of a university hospital and requiring hospitalization. Of the sample as a whole, 22.0% were diagnosed with tuberculosis and started treatment within the first 24 h after hospital admission. Diagnosis of tuberculosis and initiation of tuberculosis treatment within the first 24 h after admission were associated with being female and with an AFB-positive spontaneous sputum smear. However, there were no differences between the ≤24h group and the >24h group regarding the following outcomes: ICU admission; mechanical ventilation use; in-hospital mortality; one-year mortality; and cure rate. In addition, for the sample as a whole, the mean hospital stay was long (27.7 days), and 97% of the patients required hospitalization for more than 3 days. The fact that the mean length of hospital stay after diagnosis and initiation of treatment was 18.9 days underscores the severity of the clinical situation resulting from tuberculosis or comorbidities. 

In the present study, an AFB-positive sputum smear was significantly associated with diagnosis within the first 24 h after hospital admission. Various studies have shown that a negative sputum smear is associated with delayed diagnosis.^(^ ^7^ ^-^ ^12^ ^)^ A cross-sectional study of adults with newly diagnosed tuberculosis showed that hospitalization and delayed treatment were more likely to occur in smear-negative patients.^(^ ^12^ ^)^ In a referral hospital in Rwanda, a negative sputum smear was considered a risk factor for further health care system delay.^(^ ^9^ ^)^ Previous studies have shown that, in addition to delayed diagnosis, indicators of atypical manifestations (such as negative sputum smears) were associated with increased mortality.^(^ ^13^ ^,^ ^14^ ^)^

Being female was associated with a diagnosis of tuberculosis within the first 24 h after hospital admission. In a cross-sectional study conducted in Ethiopia,^(^ ^15^ ^)^ it was demonstrated that female patients took longer to seek medical attention than did male patients, although there was less delay in diagnosis in female patients after their entry into the health care system, a finding that is consistent with ours. Other studies have shown that being female is a risk factor for a delay in seeking medical attention (patient delay).^(^ ^16^ ^-^ ^21^ ^)^ Therefore, in the present study, female patient delay in seeking medical attention was possibly associated with a more severe and more advanced disease presentation, which raised diagnostic suspicion in the emergency room. 

Although it is recommended that the diagnosis of tuberculosis be made at primary health care clinics, a significant proportion of the population is diagnosed in public hospitals.^(^ ^10^ ^,^ ^22^ ^)^ In 2007 in Porto Alegre, 38.98% of all tuberculosis cases were reported by hospitals.^(^ ^23^ ^)^ The fact that tuberculosis is often diagnosed in hospitals is generally believed to be due to a lack of resources in primary care or the need for tests that are more specific in order to establish a diagnosis. In fact, studies have shown that delayed diagnosis is closely related to poor access to health care (which is due to the fact that health care facilities are far from where patients live), difficulties in performing tests, and the prescription of drugs other than antituberculosis drugs.^(^ ^2^ ^,^ ^11^ ^)^ However, in our study, 22% of all patients were diagnosed with tuberculosis and started tuberculosis treatment within the first 24 h after hospital admission, and positive sputum smears were associated with treatment initiation within the first 24 h after admission. This means that the diagnosis of tuberculosis was quickly established, often on the basis of sputum smear microscopy, which is a simple test that is available at primary health care clinics. Another possible explanation for this finding is the inability of primary health care professionals to recognize tuberculosis symptoms, which delays the diagnostic process. A previous study showed that cough, expectoration, and hemoptysis were less common in females than in males,^(^ ^24^ ^)^ leading physicians to suspect less of tuberculosis in the former. In addition, as discussed above, being female is usually associated with a delay in seeking medical attention, which might be due to the fact that females have to balance work and home duties or to the stigma attached to the disease; even after their entry into the health care system, difficulties in collecting sputum samples constitute an obstacle to early diagnosis in the primary care setting.^(^ ^25^ ^-^ ^29^ ^)^

The major limitation of the present study is its retrospective design; the accuracy of data collection is lower in retrospective studies than in prospective studies. Another limitation is that this was a single-center study. Nevertheless, it is important to investigate the factors associated with delayed diagnosis, because it has an impact on tuberculosis transmission. Therefore, it is crucial that the sources of delay in diagnosis be evaluated under tuberculosis control programs at hospitals. 

In conclusion, although a diagnosis of tuberculosis was established and tuberculosis treatment was initiated within the first 24 h after hospital admission in 22.0% of the new cases of tuberculosis treated in our emergency room and requiring hospitalization, the rates of in-hospital mortality, ICU admission, and mechanical ventilation use remained high. Diagnosis of tuberculosis and initiation of tuberculosis treatment within the first 24 h after admission were associated with being female and with an AFB-positive spontaneous sputum smear. Strategies for the control of tuberculosis in primary care should consider that patients who seek medical attention at hospitals arrive too late and with advanced disease. It is therefore necessary to implement active surveillance measures in the community for earlier diagnosis and treatment.
