# Supplementary material for: Tuberculosis in hospitalized patients: clinical characteristics of patients receiving treatment within the first 24 h after admission
Source: J Bras Pneumol. 2014 May-Jun;40(3):279–85. doi: 10.1590/S1806-37132014000300011 (PMC4109200; doi:10.1590/S1806-37132014000300011)
Supplement: Translation

# Translation: Tuberculose em pacientes hospitalizados: características clínicas dos pacientes que iniciaram tratamento nas primeiras 24 h de permanência hospitalar*

**Authors:** Denise Rossato Silva, Larissa Pozzebon da Silva, Paulo de Tarso Roth Dalcin

**Affiliations:** Faculdade de Medicina, Universidade Federal do Rio Grande do Sul, e Pneumologista. Serviço de Pneumologia, Hospital de Clínicas de Porto Alegre, Porto Alegre (RS) Brasil; Faculdade de Enfermagem, Universidade Federal do Rio Grande do Sul, Porto Alegre (RS) Brasil; Faculdade de Medicina, Universidade Federal do Rio Grande do Sul, e Pneumologista. Serviço de Pneumologia, Hospital de Clínicas de Porto Alegre, Porto Alegre (RS) Brasil

**Keywords:** Tuberculose, Hospitalização, Tempo para o tratamento, Medicina de emergência, Diagnóstico tardio

## Abstract

**Objetivo:**

Comparar as características clínicas e os desfechos de pacientes hospitalizados por tuberculose que iniciaram tratamento nas primeiras 24 h de permanência hospitalar com as daqueles que iniciaram tratamento após 24 h.

**Métodos:**

Estudo de coorte retrospectivo de casos novos de tuberculose com idade ≥ 18 anos que necessitaram internação hospitalar após atendimento no setor de emergência.

**Resultados:**

Foram incluídos 305 pacientes hospitalizados, dos quais 67 (22,0%) iniciaram o tratamento nas primeiras 24 h (grupo ≤24h), e 238 (88,0%) o iniciaram após (grupo >24h). Ser do sexo feminino (OR = 1,99; IC95%: 1,06-3,74; p = 0,032) e ter pesquisa de BAAR positiva no escarro espontâneo (OR = 4,19; IC95%: 1,94-9,00; p < 0,001) se associaram com o tratamento nas primeiras 24 h. Na comparação dos grupos ≤24h e >24h, a taxa de internação em UTI foi de, respectivamente, 22,4% e 15,5% (p = 0,258), enquanto a ventilação mecânica foi utilizada em 22,4% e 13,9% (p = 0,133), a taxa de óbito hospitalar foi de 22,4% e 14,7% (p = 0,189), e a taxa de cura foi de 44,8% e 52,5% (p = 0,326).

**Conclusões:**

Embora o tratamento antituberculose tenha sido iniciado rapidamente em uma proporção considerável dos pacientes hospitalizados, as taxas de mortalidade hospitalar, internação em UTI e uso de ventilação mecânica permaneceram elevadas. Estratégias para o controle de tuberculose na atenção primária devem considerar que pacientes atendidos em hospitais chegam muito tardiamente e com doença avançada, sendo necessário implementar medidas de busca ativa na comunidade para o diagnóstico e o tratamento mais precoce.

## Introdução

A tuberculose permanece um importante problema de saúde pública mundial. Estima-se que um terço da população mundial esteja infectado com o bacilo da tuberculose. Em 2011, foram estimados 9 milhões de casos novos da doença no mundo, com 1,4 milhões de mortes. O Brasil está em 22º lugar entre os 22 países com maior incidência notificada de tuberculose, apresentando 42 casos/100.000 habitantes em 2011.^(^ ^1^ ^)^

O controle da doença na comunidade depende do diagnóstico e do tratamento precoces. Apesar de os programas de controle de tuberculose preconizarem que o diagnóstico seja feito em nível de atenção básica de saúde, grande parte dos pacientes ainda é diagnosticada em hospitais. ^(^ ^2^ ^,^ ^3^ ^)^ Em Porto Alegre (RS), 39% dos casos têm sido diagnosticados em nível hospitalar.^(^ ^4^ ^)^

Um estudo prévio realizado em um hospital universitário em Porto Alegre^(^ ^5^ ^)^ mostrou que a mediana de tempo entre a admissão hospitalar e o diagnóstico de tuberculose foi de 6 dias, sendo que os principais fatores que se associaram com o atraso diagnóstico hospitalar foram a forma extrapulmonar da doença e a baciloscopia negativa do escarro. A porta de entrada desses pacientes foi sempre o serviço de emergência.

Nesse contexto, seria importante analisar as características dos pacientes com diagnóstico e início de tratamento nas primeiras 24 h de permanência hospitalar. Poderia ser conjecturado que esses pacientes não teriam necessidade de internação hospitalar e que estratégias de triagem e manejo na atenção primária poderiam ser desenvolvidas, a partir dessa análise, evitando o atendimento de emergência e reduzindo o fardo da internação hospitalar por tuberculose.

Assim, o objetivo do presente estudo foi analisar as características clínicas e os principais desfechos de pacientes hospitalizados por tuberculose que iniciaram o tratamento para a doença nas primeiras 24 h de permanência hospitalar.

## Métodos

Estudo de coorte, retrospectivo, analisando pacientes com diagnóstico de tuberculose, realizado após atendimento no Serviço de Emergência do Hospital de Clínicas de Porto Alegre (HCPA), em Porto Alegre (RS), que necessitaram internação hospitalar.

O estudo foi aprovado pelo comitê institucional de ética em pesquisa. Os autores assinaram um termo de compromisso para a utilização dos dados, responsabilizando-se por manter sigilo das informações dos pacientes estudados.

A população do estudo foi constituída por casos novos de tuberculose diagnosticados após o atendimento no Serviço de Emergência do HCPA. Foram incluídos no estudo pacientes com idade igual ou maior que 18 anos e diagnosticados como casos novos de tuberculose, de acordo com critérios de consenso.^(^ ^6^ ^)^ O diagnóstico de tuberculose pulmonar seguiu os critérios estabelecidos pelas Diretrizes Brasileiras para tuberculose^(^ ^6^ ^)^: a) detecção de BAAR pela coloração de Ziehl-Neelsen (duas amostras positivas); b) detecção de BAAR pela coloração de Ziehl-Neelsen (uma amostra positiva e uma cultura positiva para *Mycobacterium tuberculosis*); c) detecção de BAAR pela coloração de Ziehl-Neelsen e achados radiológicos compatíveis para tuberculose pulmonar; d) somente uma cultura positiva para *M. tuberculosis*; ou e) presença de achados epidemiológicos, clínicos e radiológicos compatíveis com tuberculose pulmonar, associados com uma resposta favorável ao tratamento com tuberculostáticos. O diagnóstico de tuberculose extrapulmonar foi baseado em exames clínicos e complementares de acordo com a localização da doença. Os critérios de exclusão foram os seguintes: casos notificados nos quais posteriormente houve mudança de diagnóstico e pacientes que iniciaram o tratamento antes da hospitalização.

Os pacientes foram identificados retrospectivamente através das fichas individuais de notificação de tuberculose do Sistema de Informação de Agravos de Notificação (SINAN), e os prontuários foram revisados. Em nosso hospital, a prescrição eletrônica dos medicamentos antituberculose gera automaticamente a ficha de notificação do SINAN; sendo assim, todos os pacientes que começam o tratamento puderam ser identificados.

A revisão dos prontuários foi realizada pelos investigadores, que preenchiam um questionário padronizado, incluindo os seguintes itens: dados demográficos (idade, sexo, cor e escolaridade), presença de comorbidades, tabagismo, uso de bebidas alcoólicas, uso de drogas injetáveis, uso de medicamentos imunossupressores, tuberculose prévia, forma clínica da tuberculose, sintomas na admissão, métodos de diagnóstico, esquema de tratamento utilizado, infecção pelo HIV, intervalo entre a admissão e o início do tratamento, tempo de hospitalização, admissão em UTI, necessidade e duração de ventilação mecânica, desfecho da hospitalização (alta ou óbito) e desfecho após a alta hospitalar (cura, abandono ou óbito). Os dados após a alta foram obtidos através da revisão dos prontuários, do banco de dados do SINAN e/ou através de contato telefônico com as unidades de saúde ambulatoriais responsáveis pelo tratamento do paciente após a alta.

Os dados foram digitados em uma planilha no programa Microsoft Excel^(r)^ 2010, sendo processados e analisados com auxílio do programa Statistical Package for the Social Sciences, versão 18.0 (SPSS Inc., Chicago, IL, EUA).

Foi realizada uma análise descritiva para as variáveis estudadas. Os dados quantitativos foram apresentados como média ± dp ou como mediana (intervalo interquartílico). Os dados qualitativos foram expressos em número de casos e proporção.

Para a análise estatística, os pacientes foram classificados em dois grupos: grupo ≤24h (pacientes que iniciaram tratamento para tuberculose nas primeiras 24 h de permanência hospitalar) e grupo >24h (pacientes que começaram o tratamento após as primeiras 24 h de permanência hospitalar.

A comparação das variáveis entre os grupos foi realizada utilizando o teste t para amostras independentes ou o teste U de Mann-Whitney para dados contínuos, assim como o teste do qui-quadrado para as variáveis qualitativas (quando necessário, utilizou-se a correção de Yates ou o teste exato de Fisher).

As variáveis não colineares que atingiram significância (p < 0,01) na análise univariada foram incluídas em um modelo de regressão logística binária, para cada desfecho, pelo método *forward conditional,* controlada por sexo e idade.

Todos os testes estatísticos utilizados foram bicaudais, sendo estabelecido um nível de significância de 5%.

## Resultados

Durante o período entre janeiro de 2008 e janeiro de 2011, foram incluídos no estudo 305 pacientes com diagnóstico de tuberculose.

A Tabela 1 apresenta as características dos pacientes estudados e a comparação entre os grupos de pacientes quanto ao tempo de início de tratamento. A média de idade foi de 42,0 ± 17,2 anos, e a maioria (64,6%) era do sexo masculino e da raça branca (75,4%). A forma pulmonar isolada da doença estava presente em 110 pacientes (36,1%), enquanto 143 (46,9%) apresentavam forma extrapulmonar, e 52 (17,0%) apresentavam concomitância das formas pulmonar e extrapulmonar. Eram HIV positivos 191 pacientes (62,6%). A média do tempo de internação foi de 27,7 ± 21,8 dias, sendo que nenhum paciente recebeu alta nas primeiras 24 h de permanência hospitalar, 6 pacientes (2%) receberam alta entre 24 e 48 h, e 9 (3%) receberam alta entre 24 e 72 h. A média do tempo hospitalar até início do tratamento foi de 8,8 ± 10,8 dias. O tratamento foi iniciado sempre no mesmo dia do diagnóstico. Dessa forma, os pacientes permaneceram hospitalizados, em média, mais 18,9 ± 19,1 dias após o diagnóstico e o início de tratamento. O tempo de permanência hospitalar não diferiu entre pacientes HIV positivos e negativos (p = 0,921); porém, esse foi maior nos pacientes que tinham uma ou mais comorbidades relatadas quando comparados com aqueles sem comorbidades (29,3 ± 23,1 dias vs. 23,2 ± 16,9 dias; p = 0,030).

**Tabela 1:** Características dos pacientes e comparação entre os grupos de pacientes que iniciaram tratamento para tuberculose nas primeiras 24 h de permanência hospitalar e aqueles que iniciaram após 24 h de permanência hospitalar.a

Características	Total	Grupo ≤24h	Grupo >24h	p
	N = 305	n = 67	n = 238	
Idade, anos^b^	42,0 ± 17,2	38,5 ± 16,5	42,9 ± 17,5	0,067
Idade > 60 anos	42 (13,8)	7 (10,4)	35 (14,7)	0,428
Sexo				
Masculino	197 (64,6)	34 (50,7)	163 (68,5)	0,011
Feminino	108 (35,4)	33 (49,3)	75 (31,5)	
Raça				
Branco	230 (75,4)	48 (71,6)	182 (76,5)	0,516
Não branco	75 (24,6)	19 (28,4)	56 (23,5)	
Tabagismo				
Não tabagista	162 (53,1)	33 (49,3)	129 (54,2)	0,285
Ex-tabagista	52 (17,0)	9 (13,4)	43 (18,1)	
Tabagista	91 (29,8)	25 (37,3)	66 (27,7)	
Alcoolismo	89 (29,2)	20 (29,9)	69 (29,0)	1,000
Uso de drogas ilícitas	81 (26,6)	21 (31,3)	60 (25,2)	0,397
Forma da doença				
Forma pulmonar isolada	110 (36,1)	41 (61,2)	69 (29,0)	< 0,001
Forma extrapulmonar isolada	143 (46,9)	14 (20,9)	129 (54,2)	< 0,001
Forma pulmonar e extrapulmonar	52 (17,0)	12 (17,9)	40 (16,8)	0,977
Sintomas				
Tosse	132 (43,3)	43 (64,2)	89 (37,4)	< 0,001
Emagrecimento	151 (49,5)	40 (59,7)	111 (46,6)	0,080
Sudorese noturna	74 (24,3)	24 (35,8)	50 (21,0)	0,019
Febre	190 (62,3)	39 (58,2)	151 (63,4)	0,523
Dispneia	46 (15,1)	11 (16,4)	35 (14,7)	0,879
Dor torácica	34 (11,1)	9 (13,4)	25 (10,5)	0,512
BAAR positivo no escarro espontâneo				
Sim	71 (23,3)	36 (53,7)	35 (14,7)	< 0,001
Não	93 (30,5)	14 (20,9)	79 (33,2)	
Sem escarro	141 (46,2)	17 (25,4)	124 (52,1)	
Radiografia de tórax				
Doença cavitária	33 (10,8)	15 (22,4)	18 (7,6)	0,001
Consolidação	52 (17,0)	16 (23,9)	36 (15,1)	0,134
Derrame pleural	58 (19,0)	7 (10,4)	51 (21,4)	0,065
Normal	42 (13,8)	3 (4,5)	39 (16,4)	0,015
Padrão miliar	37 (12,1)	10 (14,9)	27 (11,3)	0,405
Comorbidades				
Diabete mellitus	19 (6,2)	2 (3,0)	17 (7,1)	0,266
Insuficiência renal crônica	8 (2,6)	0 (0,0)	8 (3,4)	0,207
Transplante	7 (2,3)	0 (0,0)	7 (2,9)	0,354
Doença hepática crônica	6 (2,0)	1 (1,5)	5 (2,1)	0,744
Neoplasia	13 (4,3)	2 (3,0)	11 (4,6)	0,740
HIV	191 (62,6)	39 (58,2)	152 (63,9)	0,482
Qualquer comorbidade	224 (73,4)	47 (70,1)	177 (74,4)	0,593
Tempo de hospitalização, dias^b^	27,7 ± 21,8	19,6 ± 22,6	29,9 ± 21,1	0,001
Internação em UTI	52 (17,0)	15 (22,4)	37 (15,5)	0,258
Ventilação mecânica	48 (15,7)	15 (22,4)	33 (13,9)	0,133
Desfechos				
Óbito no hospital	50 (16,4)	15 (22,4)	35 (14,7)	0,189
Óbito após 1 ano	97 (31,8)	23 (34,3)	74 (31,1)	0,723
Cura	155 (50,8)	30 (44,8)	125 (52,5)	0,326

a Valores expressos em n (%), exceto onde indicado

b Valores expressos em média ± dp.

O tratamento para tuberculose nas primeiras 24 h de permanência hospitalar foi iniciado em 67 pacientes (22,0%). Na comparação entre os grupos, a proporção de pacientes do sexo masculino foi maior no grupo >24h do que no grupo ≤24h (68,5% vs. 50,7%; p = 0,011). A proporção de pacientes com a forma pulmonar isolada foi maior no grupo ≤24h do que no grupo >24h (61,2% vs. 29,0%; p < 0,001), enquanto a proporção da forma extrapulmonar isolada foi maior no grupo >24h do que no grupo ≤24h (54,2% vs. 20,9%; p < 0,001). A presença do sintoma tosse foi mais frequente no grupo ≤24h do que no grupo >24h (64,2% vs. 37,4%; p < 0,001), assim como a presença de sudorese noturna (35,8% vs. 21,0%; p = 0,019). A proporção de pacientes com BAAR positivo no escarro espontâneo foi significativamente maior no Grupo ≤24h do que no grupo >24h (53,7% vs. 14,7%; p < 0,001). Na avaliação radiológica convencional do tórax, a proporção de doença cavitária foi significativamente maior no grupo ≤24h do que no grupo >24h (22,4% vs. 7,6%; p = 0,001), enquanto a proporção de exames normais foi significativamente maior no grupo >24h do que no grupo ≤24h (16,4% vs. 4,5%; p = 0,015). O tempo total de hospitalização foi menor no grupo ≤24h do que no grupo >24h (19,6 ± 22,6 dias vs. 29,9 ± 21,1; p = 0,001). Os desfechos internação em UTI, ventilação mecânica, óbito no hospital, óbito após 1 ano e taxa de cura não diferiram entre os dois grupos (p > 0,05).

A Tabela 2 mostra a análise de regressão logística binária para as características associadas ao início do tratamento para tuberculose nas primeiras 24 h de permanência hospitalar. Observa-se que as variáveis sexo feminino (OR = 1,99; IC95%: 1,06-3,74; p = 0,032) e pesquisa de BAAR positiva no escarro espontâneo (OR = 4,19; IC95%: 1,94-9,00, p<0,001) se associaram de forma independente com o início de tratamento nas primeiras 24 h de permanência hospitalar.

**Tabela 2:** Regressão logística binária para características associadas ao início do tratamento para tuberculose nas primeiras 24 h de permanência hospitalar.

Variável	b	Wald	Significância	OR	IC95%
Idade	0,01	2,26	0,133	1,02	1,00-1,03
Sexo feminino	0,69	4,60	0,032	1,99	1,06-3,74
Forma pulmonar isolada	0,73	2,88	0,090	2,08	0,89-4,83
Forma extrapulmonar isolada	0,04	0,005	0,942	1,04	0,36-3,00
Tosse	0,34	0,94	0,333	1,40	0,71-2,76
Sudorese noturna	0,41	1,30	0,254	1,50	0,75-3,00
BAAR positivo no escarro espontâneo	1,43	13,37	< 0,001	4,19	1,94-9,00
Doença cavitária	−0,10	0,05	0,828	0,90	0,36-2,26
Derrame pleural	−0,50	1,00	0,317	0,61	0,23-1,62
Radiografia de tórax normal	−0,57	0,63	0,428	0,57	0,14-2,31
Constante	−0,57	0,26	0,611	0,57	-

## Discussão

O presente estudo de coorte retrospectivo avaliou os casos novos de tuberculose atendidos no serviço de emergência de um hospital universitário e que tiveram necessidade de internação hospitalar. Foi identificado que uma proporção de 22,0% dos pacientes teve o diagnóstico de tuberculose e o início do tratamento nas primeiras 24 h de permanência hospitalar. As variáveis que se associaram com esse grupo de diagnóstico e tratamento nas primeiras 24 h foram o sexo feminino e a pesquisa de BAAR positiva no escarro espontâneo. Entretanto, a despeito do rápido diagnóstico e tratamento hospitalar nesse grupo de pacientes, os desfechos internação em UTI, ventilação mecânica, óbito no hospital, óbito após 1 ano e taxa de cura não diferiram daqueles dos pacientes com diagnóstico e tratamento depois de 24 h de permanência hospitalar. Ainda, para o grupo total de pacientes, a média do tempo de internação foi longo (27,7 dias), e 97% dos pacientes necessitaram permanecer mais de 3 dias internados. O fato de os pacientes terem permanecido hospitalizados, em média, por mais 18,9 dias após o diagnóstico e o início de tratamento reforça a gravidade da situação clínica em decorrência da tuberculose ou das comorbidades.

No presente estudo, a pesquisa de BAAR positiva no escarro foi significativamente associada com o diagnóstico nas primeiras 24 h de internação. Diversos estudos já demonstraram que a baciloscopia de escarro negativa é associada com o atraso no diagnóstico.^(^ ^7^ ^-^ ^12^ ^)^ Um estudo transversal de pacientes adultos com diagnóstico recente de tuberculose demonstrou que pacientes com baciloscopia negativa tinham uma maior probabilidade de serem hospitalizados e de ter atraso no início do tratamento.^(^ ^12^ ^)^ Em um hospital de referência em Ruanda, a baciloscopia de escarro negativa foi considerada um fator de risco para um atraso maior relacionado ao sistema de saúde.^(^ ^9^ ^)^ Estudos prévios evidenciaram que, além do atraso no diagnóstico, indicadores de manifestações atípicas, como a baciloscopia negativa, estariam associados com o aumento de mortalidade.^(^ ^13^ ^,^ ^14^ ^)^

O sexo feminino se associou com o diagnóstico nas primeiras 24 h de internação hospitalar. Em um estudo transversal realizado na Etiópia,^(^ ^15^ ^)^ foi demonstrado que as mulheres demoravam mais tempo que os homens para buscar atendimento, mas tinham um menor atraso no diagnóstico depois de entrarem no sistema de saúde, o que corrobora nosso achado. Outros estudos demonstraram que o sexo feminino é um fator de risco para o atraso na busca por atendimento médico (atraso do paciente).^(^ ^16^ ^-^ ^21^ ^)^ Dessa forma, é possível que, no presente estudo, a procura tardia do sistema de saúde pelas mulheres esteja associada com uma apresentação mais grave e mais avançada da doença, facilitando a suspeita diagnóstica ainda na sala de emergência.

Apesar de ser preconizado que o diagnóstico da tuberculose seja oferecido em nível primário de atenção em saúde, uma parcela significativa da população ainda é diagnosticada em hospitais públicos.^(^ ^10^ ^,^ ^22^ ^)^ Em Porto Alegre, no ano de 2007, 38,98% dos casos foram notificados pelos hospitais.^(^ ^23^ ^)^ Considera-se muitas vezes que essa procura elevada pelo diagnóstico em hospitais seja devida à falta de recursos na rede básica de saúde ou à necessidade de exames mais especializados para o diagnóstico. De fato, estudos demonstram que o atraso no diagnóstico é intimamente relacionado com o pobre acesso ao sistema de saúde pela distância dos serviços de saúde, dificuldades na realização de exames e prescrição de outras medicações que não tuberculostáticos.^(^ ^2^ ^,^ ^11^ ^)^ Entretanto, em nosso estudo, 22% dos pacientes tiveram o diagnóstico e o início de tratamento nas primeiras 24 h de internação, e a baciloscopia de escarro positiva foi um fator associado com o início de tratamento nas primeiras 24 h. Ou seja, o diagnóstico foi rápido e muitas vezes através da baciloscopia de escarro, exame simples e disponível nos locais de atenção primária. Outra possível explicação para esse achado seria a falta de reconhecimento dos sintomas de tuberculose pelos profissionais da atenção primária, que acabaria retardando o processo diagnóstico. Um estudo prévio demonstrou que tosse, expectoração e hemoptise foram menos comuns em mulheres do que em homens,^(^ ^24^ ^)^ o que poderia levar os médicos a suspeitar menos de tuberculose nessas pacientes. Além disso, como anteriormente exposto, o sexo feminino costuma estar associado com o atraso na busca de atendimento, por questões relacionadas à dupla jornada de trabalho ou ao estigma da doença, e mesmo quando já no sistema de saúde, dificuldades na coleta de escarro podem impedir um diagnóstico mais precoce na rede básica de saúde.^(^ ^25^ ^-^ ^29^ ^)^

A principal limitação do presente estudo é o seu delineamento retrospectivo, já que acurácia da coleta de dados é sempre menor que nos estudos prospectivos. Outra limitação é que o estudo envolveu apenas um centro. Apesar disso, o estudo dos fatores associados com um diagnóstico mais tardio é importante, uma vez que isso tem um impacto na transmissão da tuberculose. Portanto, a identificação das fontes de atraso no diagnóstico é um ponto crucial para ser avaliado nos programas de controle hospitalar da tuberculose.

Concluindo, o presente estudo demonstrou que embora uma proporção de 22,0% de casos novos de tuberculose atendidos em um serviço de emergência e com necessidade de internação hospitalar tiveram o diagnóstico de tuberculose e iniciaram tratamento nas primeiras 24 h de permanência hospitalar, a mortalidade e as taxas de internação em UTI e de ventilação mecânica permaneceram elevadas. O sexo feminino e a pesquisa de BAAR positiva no escarro espontâneo se associaram com o diagnóstico e o tratamento nas primeiras 24 h. Estratégias para o controle de tuberculose na atenção primária devem considerar que os pacientes atendidos no hospital chegam muito tardiamente e com doença avançada, sendo necessário antecipar medidas de busca ativa na comunidade para um diagnóstico mais precoce.
